# Range contraction of snow leopard (*Panthera uncia*)

**DOI:** 10.1371/journal.pone.0218460

**Published:** 2019-08-01

**Authors:** Tariq Mahmood, Ayesha Younas, Faraz Akrim, Shaista Andleeb, Abdul Hamid, Muhammad Sajid Nadeem

**Affiliations:** 1 Department of Wildlife Management, Pir Mehr Ali Shah Arid Agriculture University, Rawalpindi, Pakistan; 2 Department of Zoology, University of Kotli, Kotli, Azad Jammu and Kashmir, Pakistan; 3 Department of Zoology, Pir Mehr Ali Shah Arid Agriculture University, Rawalpindi, Pakistan; Sichuan University, CHINA

## Abstract

Snow Leopard (*Panthera uncia*) is native to mountain ranges of Central and South Asia, where it occurs from 3,000–4500 m elevation. The species is enlisted as “Endangered” by IUCN and its populations are reportedly declining. In the current study, we compared the past and current distribution ranges of the species using spatial analysis. We used Quantum Geographical Information System (QGIS) software to reconstruct and quantify its past distribution range and compare with its current one. Snow leopard was found more widely distributed in the past having a distribution range of approximately 10.47 million km^2^ against the current 3.20 million km^2^. Range contraction of the species approximates 69%. A total of 719 terrestrial protected areas of Asia (out of total 7209) had this species in the past whereas at current, only 311 protected areas support this species. The results emphasize escalating conservation efforts to save its remaining distribution range.

## Introduction

The snow leopard is currently distributed in 12 different countries, including Afghanistan, Bhutan, China, India, Kazakhstan, Kyrgyzstan, Mongolia, Nepal, Pakistan, Russia, Tajikistan and Uzbekistan [1]. The species has been classified as “Endangered” under C1 in the IUCN list of threatened species (IUCN, 2015). Its documented distribution range extends from the Himalayas in the South, across the Qinghai-Tibet Plateau, and the mountains of Central Asia, to the mountains of Southern Siberia in the North. It is present in Atlai, Sayan, Tien Shan, Kunlun, Pamir, Hindu Kush, Karakoram, and Himalayan Ranges and in smaller isolated mountains of Gobi Region [2]. Large parts of the snow leopard’s range are not surveyed or surveyed about 10 or 20 years ago, especially for its distribution and population estimates. Another difficulty in documenting its distribution is the fact that it is mostly present across international borders [3].

The estimated habitat of snow leopard is variable; however, its estimated potential habitat includes 1.245 million km^2^ and its global population approximating 5329–6140 individuals [4]. Whereas, another study reports its potential habitat as 18, 46000 km^2^ and its global population approximating 4360–7240 individuals [5], still some other reports document its potential habitat as being 18. 35 million km^2^ and its global population of 4510–7350 individuals [6]. The potential habitat of snow leopard using GIS was estimated to be at 3 million km^2^ with 6% falling within existing network of protected areas [3].

Snow Leopard normally inhabits rugged ranges and is associated through most of the range with arid and semi-arid shrub lands, grasslands or steppes [7]. It generally occurs at elevations ranging between 3,000 and 4,500 m, which may occasionally go up to 5, 500 m in the Himalayas. On the other hand, the species may also occur at much lower elevations such as from 560 m to 1500 m as in some areas of Russia. The species has got a superb camouflage for its mountain environment of bare rocks and snow, being whitish-grey in color. Its adaptations for high altitude life include, an enlarged nasal cavity, shortened limbs, well-developed chest muscles, long hair and a tail which may be up to 1m long, and approximately 75–90% of the head-body length [7,8]. It is adapted for living in freezing cold environment, for this purpose, its body is stocky, fur is thick and woolly, and ears are small and rounded, which minimize the heat loss.

A total of 24% species of mammals are considered threatened and substantial range contractions have occurred among the species whose global conservation status is assessed as “Least Concerned”. Ranges of some large mammals have got greatly reduced due to human activities through direct habitat alteration or direct exploitation [9,10]. More recently the distribution ranges some mammalian species have been estimated, for example, Sukumar [11] estimated historical distributional range of Asian elephant (*Elephas maximus*) as over 9 million km^2^, similarly, Sanderson et al. [12] and Walston et al. [13] reported that Asiatic tiger has lost over 93% of its historical distribution range.

Although some recent studies such as in Eastern Pamir[14], Bhutan [15–17], India [18,19], Kyrgyzstan [20], China [21,22] and Mongolia [23] have focused on snow leopard distribution and abundance, and these contribute to our understanding of snow leopard population estimates, but none of these have targeted and investigated the range contraction of the species. Since information on historical or past distribution range of snow leopard is scanty, and the species is listed as “Vulnerable” [24] throughout its range and “Critically Endangered” in Pakistan [25], current study, therefore, aimed at reconstructing and quantifying the past and current distribution ranges of the snow leopard.

## Methods

### Data collection

Data about past or historical distribution of snow leopard in Asia were collected by searching or retrieving all kinds of published records including scientific journals, books, newspaper articles, and also by personal communication. We considered ‘historical distribution’ as the natural occurrence of a species anytime in the past, whereas, the “current distribution” of snow leopard means the one obtained from the latest IUCN Red List of Threatened species [24].

The past distribution of snow leopard was reconstructed using available information from published and unpublished resources, including peer-reviewed journal articles, books, unpublished research theses, and newspaper articles. Geographic information about locations with known presence of the target species outside the current range was recorded together with information on the time when the species was present, the strength of evidence (whether the information was based on paleontological remains, hunting, direct sighting, or accounts from local communities), and other relevant ecological information (vegetation type, altitude, when available).

### Data processing and analysis

The locations of snow leopard’s occurrence in the past were fed in Google Earth software to obtain the latitude-longitude geographic coordinates in the form of KML (Keyhole Markup Language) files, which were later exported to Quantum Geographic Information Systems (QGIS; Quantum GIS Development Team, 2012) and converted and saved as shape files. The result was a layer of points with known past or historical presence of the species. For each location point, we created a buffer of the size of the known average home range size of the snow leopard (50 km^*2*^). The created buffers contained some non-terrestrial areas as well, for which, we had to clip away those non-terrestrial areas (large inland aquatic systems) using “global topographical data” layer of the earth downloaded from http://www.webgis.com/terr_world.html. Finally, we used QGIS’ toggle editor to fill up gaps within the resulting distribution ranges based on previously existing historical maps and ecological factors. After reconstruction of the past distribution range polygon, quantification of the total past or historical range was done by using same QGIS.

The current distribution of the snow leopard was retrieved from IUCN’s Red List of Threatened Species website (http://www.iucnredlist.org/technical-documents/spatial-data) as a shape file document; these distributions were recently published [24], therefore our ‘current range’ represents the best knowledge about the distribution of our target species at the moment. In the end, quantification of the size of the historical range and current distribution range of snow leopard were done.

### Range quantification

The total area of the past and the current distribution ranges of snow leopard was estimated/quantified by summation of the individual polygons using QGIS software. A comparison was then made between the total areas of past or historical versus current ranges to estimate the range contraction/expansion for the species under study. The percent range difference (range lost) was estimated using the quantification data generated from the historical and current distribution ranges.

### Protected Areas and snow leopard distribution range

“We mapped the historical and present occurrence of snow leopards in protected areas (PAs) using the “World Database on Protected Areas (https://www.protectedplanet.net/)” [26]. Change in snow leopard distribution was documented and ecological and anthropomorphic variables were analyzed to characterize protected areas where snow leopards survived or were extirpated”.

## Results

### Past distribution records of snow leopard

**Fig 1:**
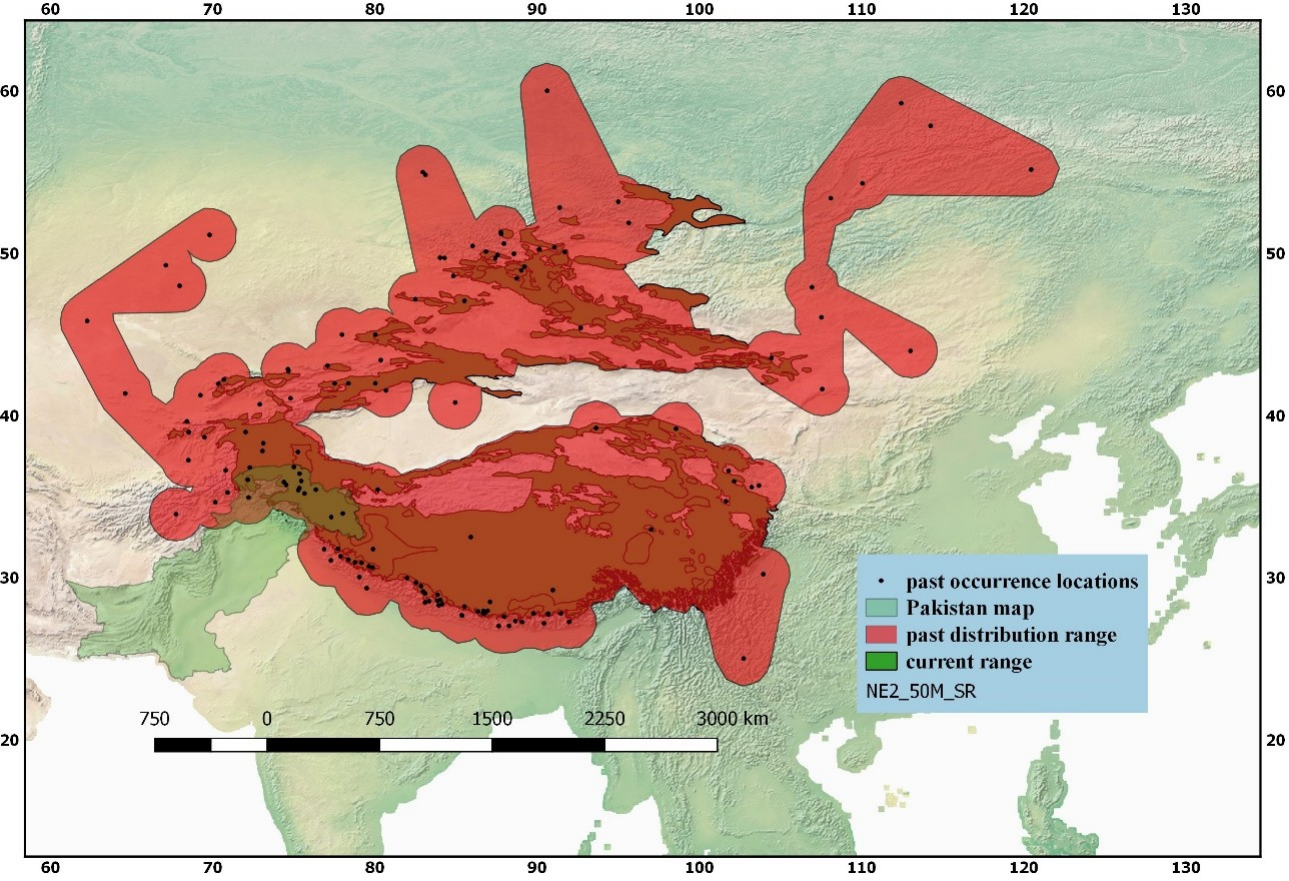
GIS-based distribution map of snow leopard (*Panthera uncia*) showing its past distribution range reconstructed from the available data on its past occurrence and its comparison with the current distribution range of the species retrieved as spatial data from the IUCN Red List of threatened species.

The past distribution records of Snow leopard (*Panthera uncia*) collected during the current study showed that the cat species was distributed in many areas of the twelve (12) different range countries including India, Pakistan, Nepal, Bhutan, Afghanistan, Russia, China, Mongolia, Uzbekistan, Kyrgyzstan, Kazakhstan, and Tajikistan (Fig 1). The oldest record collected about its distribution was of the year 1837, and that was about occurrence of snow leopard in Siberia, and Russia. During data collection about snow leopard distribution, some unusual or odd distribution records (New Guinea, Oceania, Argut River, Altai) were also found which could not be verified from any other source, hence they were not included in the final analysis.

### Reconstruction and quantification of past distribution range

The past distribution range of snow leopard was reconstructed by creating buffers around the location points utilizing the collected data using Quantum Geographic Information System (QGIS; Version 2.18.6). Buffer size was established using information about home range size of snow leopard. Various records about the home range size of the species were found in literature such as in Mongolia, it was estimated to be, 61 km^2^, 142 km^2^, 14km^2^ and 58 km^2^, respectively. We, in the current study, used average home range size of 50 km^2^ to create buffers around the location points of the past distribution of the species. However, non-terrestrial parts of the buffers were excised out and only terrestrial areas were included in the analysis.

We reconstructed the past distribution range of snow leopard using QGIS’ toggle editor to fill up the gaps within the resulting distribution ranges based on previously existing historical maps and ecological factors. After reconstruction of the past distribution range polygon, quantification of the total past or historical range was done using geometry tools option in the QGIS to measure the distribution polygon created (Fig 1; Table 1). Results showed that the estimated area of the past distribution range of snow leopard was approximately 10.471 million km^2^.

**Table 1:** Quantification of past and current distribution ranges of the snow leopard (*Panthera uncia*) using QGIS software.

Range (million km^2^)	Past distribution	Current distribution range (million km^2^)	Range contraction (million km^2^)	% Range contraction
Current study	10.471	3.20	7.271	69.44%
GSLEP (2013)	10.471(current study)	1.77	8.701	83.09%

Percent Reduction = Past Range ÷ Current Range × 100

### Current distribution range and its quantification

Spatial data about current distribution range of snow leopard obtained from the IUCN Red List of Threatened species (2015) was utilized for analysis of current distribution of the species; the distribution range was mapped and quantified using QGIS software which showed an area approximately 3.20 million km^2^ is occupied by the species at current (Table 2; Fig 1). Some earlier published records on estimates of distribution range of snow leopard have been shown in Table 2 for comparison with the current study estimates.

**Table 2:** Details of some of the major Protected Areas (PA’s) of Asia included in the past and current distribution ranges of the snow leopard (*Panthera uncia*).

Protected Areas	Category	Country	Area (km2)	Past Range	Current Range
1. Gissarsky state Nature reserve	State Nature reserve	Uzbekistan	1628.76	Yes	Yes
2. Khangchendzonga National Park	National Park	India	1784	Yes	Yes
3. Langtang National Park	National Park	Nepal	1710	Yes	Yes
4. Sagarmatha National Park	National Park	Nepal	1148	Yes	Yes
5. Khunjerab National Park	National Park	Pakistan	2269.13	Yes	Yes
6. Pamir-e-Buzurg Wildlife Reserve	Wildlife Reserve	Afghanistan	679.38	Yes	Yes
7. Dachigam National Park	National Park	India	171.25	Yes	Yes
8. Hemis National Park	National Park	India	4100	Yes	Yes
9. Jigme-Dorji National Park	National Park	Bhutan	4349.946	Yes	Yes
10. Sheikh Buddin National Park	National Park	Pakistan	195.4	Yes	No
11. Astore Wildlife Sanctuary	Wildlife Sanctuary	Pakistan	414.72	Yes	Yes
12. Shey-Phoksundo National Park	National Park	Nepal	3555	Yes	Yes
13. Royal Manas National Park	National Park	Bhutan	1022.838	Yes	No
14. Kurgal’ Dzhinskiy State Nature Reserve	State Nature Reserve	Kazakhzstan	2431.38	Yes	No
15. Aksu-Dzhabagly State Nature Reserve	State Nature Reserve	Kazakhzstan	750.94	Yes	Yes
16. Annapurna Conservation Area	Conservation Area	Nepal	7629	Yes	Yes
17. Dhorpatan Hunting Reserve	Hunting Reserve	Nepal	1325	Yes	Yes
18. Ghamot Game Reserve	Game Reserve	Pakistan	272.83	Yes	Yes
19. Argham Basti Wildlife Sanctuary	Wildlife Sanctuary	Pakistan	298.66	Yes	Yes
20. Kaziranga National Park	National Park	India	849.79	Yes	No
21. Royal Chitwan National Park	National Park	Nepal	932	Yes	No
22. Band-e-Amir Provisional National Park	Provisional National Park	Afghanistan	596.5	Yes	No
23. Alma-Atinskiy State Nature Reserve	State Nature Reserve	Kazakhstan	733.42	Yes	Yes
24. Besh-Aral State Nature Reserve	State Nature Reserve	Kazakhstan	632	Yes	Yes
25. Marakol’skiy State Nature Reserve	State Nature Reserve	Kazakhstan	750.4	Yes	Yes
26. Chatkalskiy State Nature Reserve	State Nature Reserve	Uzbekistan	713.72	Yes	Yes
27. Zaaminskiy State Nature Reserve	State Nature Reserve	Uzbekistan	536.94	Yes	Yes
28. Kedarnath Sanctuary	Sanctuary	India	975.24	Yes	Yes
29. Ar-Toul Hunting Reserve	Hunting Reserve	Mongolia	7993.6	Yes	No

### Range contraction

Results of quantification of the both distribution ranges (past and current) of snow leopard showed that the species had a much broader distribution range (10.471 million km^2^) in the past (Table 1; Fig 1). However, at current its distribution range is much smaller (3.20 million km^2^). A comparison of the past and current distribution ranges of the species, thus, showed a considerable (approximately 69.44%) range contraction (Table 1) including an area equal to 7.271 million km^2^). If we used current range estimates (1.77 million km^2^) of GSLEP (2013), the range contraction further becomes severe getting equals to approximately 83% (Tables 1 and 3).

**Table 3:** Previous records depicting estimates of current distribution range of snow leopard (Taken from Snow Leopard Network, 2014).

Estimate (km^2^)	Source
1, 230,000	Fox (1989)
1,835,000	Fox (1994)
3,024,728	Hunter and Jackson (1997)
1,003,608	Beijing (2008)
219,489	Beijing (2008)
1,535,116	Beijing (2008)
2,758,213	Beijing (2008)
1,200,000–1,600,000	Jackson et al. (2010)
1,776,000	GSLEP (2013)

### Protected Areas (PA’s) of Asia and past and current distribution of snow leopard

**Fig 2:**
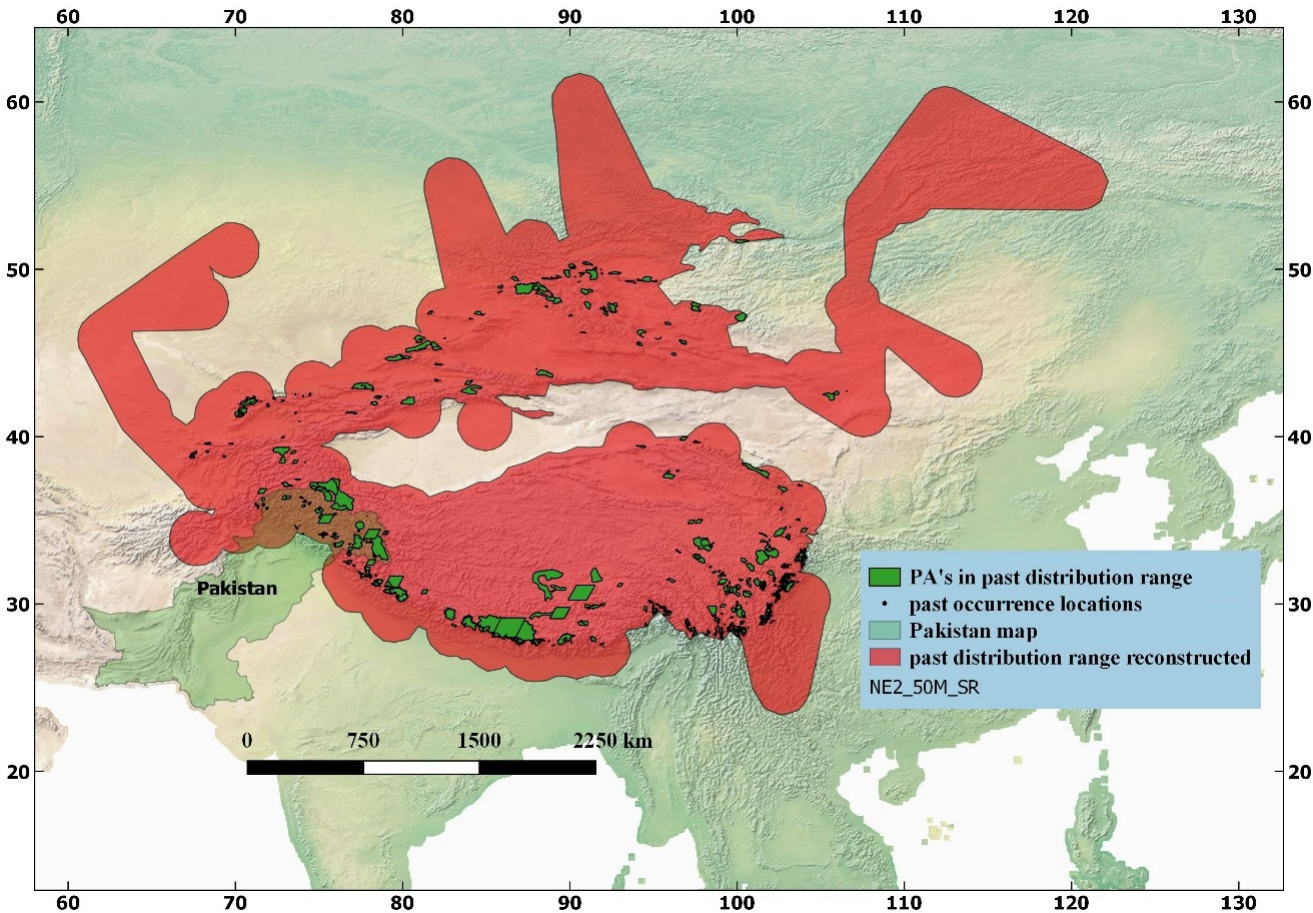
GIS-based map of snow leopard (*Panthera uncia*) in relation to Protected Areas (PA’s) of Asia of various sizes (N = 719) that were present in the past distribution range of snow leopard. Spatial data on PA’s was retrieved from the “Protected Planet” website and modified in Quantum Geographical Information System.

Distribution (past and current) of snow leopard was also studied in Protected Areas (PA’s) of various types in the range countries (Fig 2). For this purpose, spatial data about protected areas of Asia was downloaded from WDPA (World Data on Protected Areas), Protected Planet (http://www.wdpa.org). Only terrestrial PA’s of Asia were included in current analysis while the marine PA’s were removed from the data layer. Total numbers of PA’s of Asia (excluding marine PA’s) were N = **7209**, and the largest protected area was, Annapurna Conservation Area (size = 7629 km^2^), Nepal. Spatial analysis showed that out of total (N = 7209) PA’s of Asia, n = 719 PA’s of various sizes were included in the past distribution range of the species (Fig 2; Table 2). Some major PA’s in this context included Annapurna Conservation Area (Nepal), Sagarmatha National Park (Nepal), Jigme Dorji National Park (Bhutan), Hemis National Park (India), Sheikh Buddin National Park (Pakistan), Zaaminskiy State Nature Reserve (Uzbekistan) and Kurgal’ Dzhinskiy State Nature Reserve (Kazakhstan) and others. Protected Areas of Pakistan that fell in the past range of snow leopard included Khunjerab National Park, Sheikh Buddin National Park and Astore Wildlife Sanctuary (Fig 2; Table 2).

**Fig 3:**
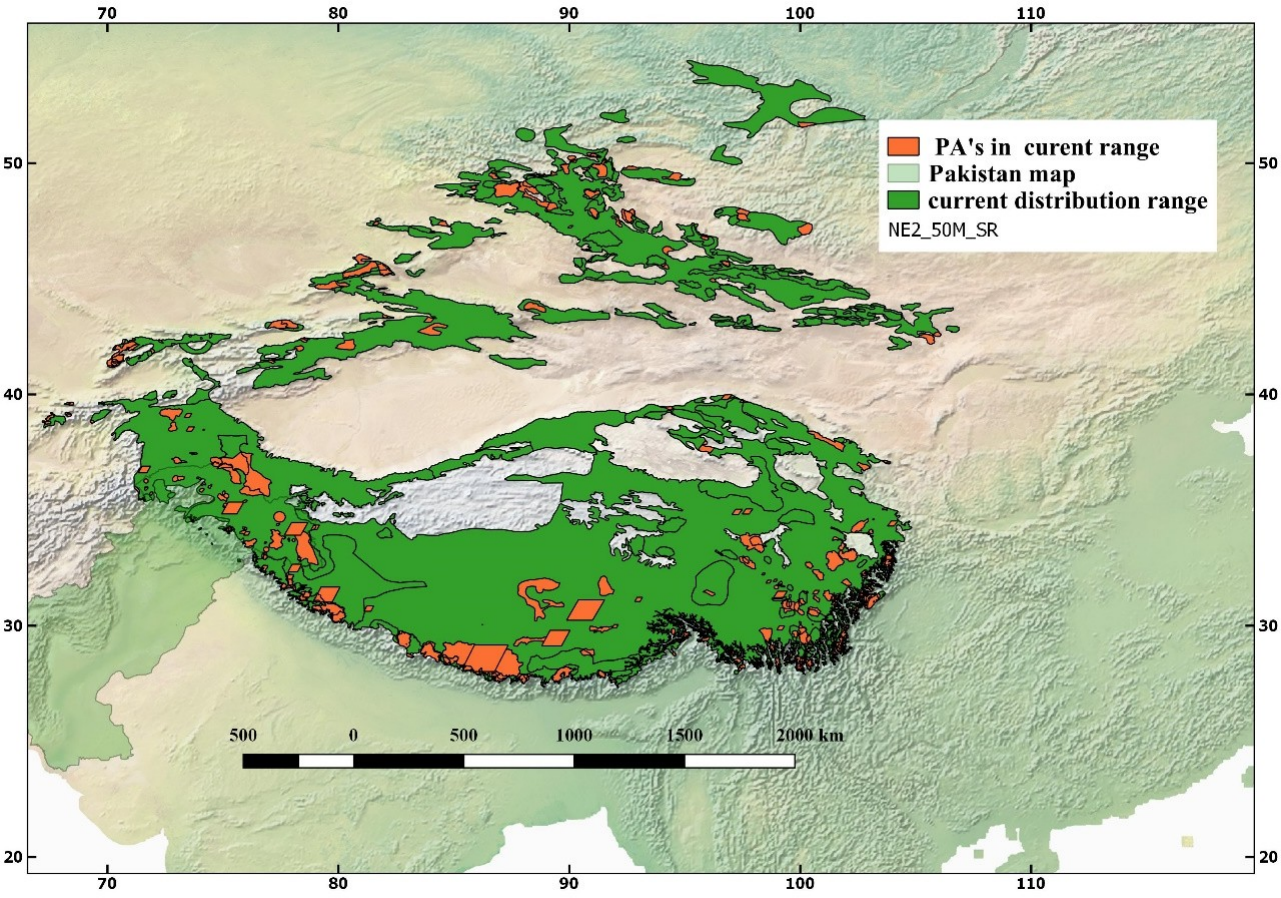
A map of snow leopard (*Panthera uncia*) in relation to Protected Areas (PA’s) of Asia (N = 311) that are included in the current distribution range of the species. Spatial data on PA’s was retrieved from the “Protected Planet” website and modified in Quantum Geographical Information System.

Similarly, a total of n = 311 (out of N = 7209 PA’s of Asia), are included in the current distribution range of Snow Leopard (Fig 3; Table 2), as against n = 719 PA’s in the past distribution range of the species (Fig 2). Some of the major PA’s present in the current distribution range of snow leopard include Khangchendzonga National Park (India), Pamir-e-Buzurg Wildlife Reserve (Afghanistan), Royal Manas National Park (Bhutan), Dhorpatan Hunting Reserve (Nepal) and Wanba Nature Reserve (China).

A comparison of the past and current distribution ranges of snow leopard indicates that not only the species has vanished from non-protected areas but also many of the Protected Areas (n = 408; 56%) have lost the species where it was previously present (Table 4; Fig 2).

**Table 4:** Details of some of prominent Protected Areas (PA’s) of Asia that have lost snow leopard (*Panthera uncia*) at the current.

Name of Protected Areas	Category	Country	Area covered	Past Range	Current Range
1. Band-e-Amir Provisional National Park	National Park	Afghanistan	596.5	Yes	No
2. Royal Bardia National Park	National Park	Nepal	968	Yes	No
3. Kurgal’ Dzhinskiy State Nature Reserve	State Nature Reserve	Kazakhstan	2431.38	Yes	No
4. Pakhui Sanctuary	Sanctuary	India	861.95	Yes	No
5. Ar-Toul Hunting Reserve	Hunting Reserve	Mongolia	7993.6	Yes	No
6. Hastinapur Sanctuary	Sanctuary	India	2073	Yes	No
7. Kala Chitta Game Reserve	Game Reserve	Pakistan	1326.11	Yes	No
8. Alakolskiy State Nature Reserve	State Nature Reserve	Kazakhstan	1000	Yes	No
9. Surkhanskiy State Nature Reserve	State Nature Reserve	Uzbekistan	553.52	Yes	No

## Discussion

Range contraction precedes the loss of populations and species. Understanding and quantifying range contractions of threatened megafauna can help to develop effective conservation and restoration policies [27]. Some earlier attempts by researchers have shown substantial declines in home ranges of some mammalian fauna at different geographical and temporal scales [9,27]. On a global scale, less than 21% of earth’s terrestrial surface is estimated to have an intact assemblage of large (> 20 kg) mammals [28]. The Indo-Malayan region, an area rich in diversity and large mammals [29] is considered to be particularly sensitive to mammal decline [9,30], retaining an intact large-mammal assemblage in just 1% of its surface [28]. Previous studies have focused on range contractions over periods ranging from a few decades [31] to a few centuries [27,28,32]. Larger species–whether among herbivores or carnivores–had larger original distribution ranges and have also suffered the most acute range reductions. This contrasts with the results of Ceballos and Ehrlich [9], who found no effect of body size in the range contraction patterns of 173 mammals’ species across the globe.

Animals have got suitable environment and set distributional records. The snow leopard (*Panthera uncia*) is, generally, distributed at higher elevations and its range being limited to Asian continent only. The species in some parts of its range is also sympatric with other species like common leopard, and ibex and few others, which share the same habitat and living conditions; however, snow leopard has an exception of being found at extremely high elevations. However, it is worth mentioning that across much of its range, snow leopards are dependent on ibex (as major wild prey), which contributes substantial portion of its wild prey in its diet composition in northern areas of Pakistan [33]. Different researchers have focused on populations and distribution of snow leopard in specific countries, but no records so far exist about the estimates of its total distribution range description in Asia. Obviously, there is a limitation in calculating its overall range, since the past published data or records of snow leopard occurrence are not easily available or accessible as such. We, in the current study, have reconstructed the past distribution range of snow leopard using all sorts of diverse possible information and extracting the geographic information about locations with known presence of snow leopards outside their current range, along with associated information on the time when the species was present, the strength of evidence and other relevant ecological information (vegetation type, altitude, etc) and then data were finally analyzed.

Reconstructed past distribution range of snow leopard, in the current study, has been found much wider, spanning over an area of approximately 10.47 million km^2^. In comparison, the current distribution range of snow leopard estimated in the current study by using spatial data obtained from IUCN Red list of Threatened Species, 2015 is approximately 3.20 million km^2^. According to Snow Leopard Network [2], “much of the snow leopard’s current distribution is located along contentious international borders, adding to the difficulty of reliably establishing the species’ current status and distribution. Depicting the current distribution of the snow leopard at a fine scale is, therefore, not straightforward. These factors partly explain the wide range in estimates of global range size, varying from 1.2 million to over 3 million km^2^”. In a similar study, the range of snow leopard was estimated to be more than 3 million km^2^ based on elevational analysis, with much of it occurred in Mongolia and Tibetan Plateau of China [25]. In this context, the estimates of current distribution range of snow leopard in the current study seem logical. A comparison of the past and current distribution ranges of snow leopard has shown a huge difference of approximately 10 million km^2^, which indicates that current range of snow leopard, has become substantially reduced whereby the range contraction is estimated to be approximately 69%. Although a few earlier studies have estimated potential range of snow leopard in some countries, however, no earlier estimates of snow leopard’s historical distribution range, like the one in the current study, are available for comparison. However, few earlier studies on some other carnivore species have already indicated substantial range contraction in many species including such as by Sanderson et al. [12] and Walston et al. [13] estimated and reported that Asiatic tiger had lost 93% of its past distribution range.

Protected Areas play vital role in the long-term conservation of nature with associated ecosystem services and cultural values. The IUCN recognizes six different categories of PA’s world that provide for wildlife’s diversity in safeguarding species and habitats. Every species evolves from million years of change and contribute to extra-ordinary living creatures on Earth [34]. In the current study, spatial data about protected areas (PA’s) of Asia downloaded from the website “Protectedplanet.net” have shown that a total of 408 PA’s of Asia have already lost snow leopard where the species was present in the past. So, in this regard, range contraction for snow leopard also involves both protected and non-protected areas.

Throughout the world there is an increasing interest in restoring ecological processes, including the recovery of long missing wildlife and their associated ecological processes that once controlled them [35]. With so much range lost by Asian megafauna, conservation objectives should focus not only in protecting extant populations–the main priority–but also on restoring lost populations and the ecological role of megafauna. Our maps can be used as a tool to prioritize rewilding projects in Asia. Examples of successful rewilding efforts include the reintroduction of beavers throughout much of Europe [36] or wolves in parts of North America [37]. Rewilding projects exist throughout the world but are more common in temperate latitudes. In addition, for top carnivores, where the loss of habitat has contributed towards the population decline, important factor could be to conserve the prey species of the carnivore (like snow leopard) to ensure the availability of the wild prey in its habitat, so as to reduce depredation of snow leopard on livestock. Resultantly it will reduce the human-snow leopard negative interaction in the range area. These measures can improve the habitat that is left, and in turn will increase the population size of the snow leopard.
